# Global Levels of Histone Modifications in Peripheral Blood Mononuclear Cells of Subjects with Exposure to Nickel

**DOI:** 10.1289/ehp.1104140

**Published:** 2011-10-24

**Authors:** Adriana Arita, Jingping Niu, Qingshan Qu, Najuan Zhao, Ye Ruan, Arthur Nadas, Yana Chervona, Fen Wu, Hong Sun, Richard B. Hayes, Max Costa

**Affiliations:** 1 Department of Environmental Medicine, New York University School of Medicine, New York, New York, USA; 2 Lanzhou University School of Public Health, Lanzhou, China; 3 New York University Cancer Institute, New York, New York, USA

**Keywords:** epigenetics, H3K4 trimethylation, H3K9 acetylation, H3K9 dimethylation, histone modifications, interindividual variation, intraindividual variation, nickel, nickel refinery workers

## Abstract

Background: Occupational exposure to nickel (Ni) is associated with an increased risk for lung and nasal cancers. Ni compounds exhibit weak mutagenic activity, cause gene amplification, and disrupt cellular epigenetic homeostasis. However, the Ni-induced changes in global histone modification levels have only been tested *in vitro*.

Objective: This study was conducted in a Chinese population to determine whether occupational exposure to Ni is associated with alterations of global histone modification levels and to evaluate the inter- and intraindividual variance of global histone modification levels.

Method: Forty-five subjects with occupational exposure to Ni and 75 referents were recruited. Urinary Ni and global H3K4 trimethylation, H3K9 acetylation, and H3K9 dimethylation levels were measured in peripheral blood mononuclear cells (PBMCs) of subjects.

Results: H3K4me3 was elevated in Ni-exposed subjects (0.25% ± 0.11%) compared with referents (0.15% ± 0.04%; *p* = 0.0004), and H3K9me2 was decreased (Ni-exposed subjects, 0.11% ± 0.05%; referents, 0.15% ± 0.04%; *p* = 0.003). H3K4me3 was positively (*r* = 0.4, *p* = 0.0008) and H3K9ac was negatively (*r* = 0.1, *p* = 0.01) associated with urinary Ni. Interindividual variances of H3K4me3, H3K9ac, and H3K9me2 were larger compared with intraindividual variance in both exposure test groups, resulting in reliability coefficients (an estimate of consistency of a set of measurements) of 0.60, 0.67, and 0.79 for H3K4me3, H3K9ac, and H3K9me2, respectively, for Ni-exposed subjects and of 0.75, 0.74, and 0.97, respectively, for referent subjects.

Conclusion: The results of this study indicate that occupational exposure to Ni is associated with alterations of global histone modification levels and that measurements of global levels of histone modifications are relatively stable over time in human PBMCs.

Nickel (Ni), one of the most abundant transition metals in the earth’s crust, is widely distributed in the environment. Natural sources of atmospheric Ni include dusts from volcanic emissions and the weathering of rocks and soils (Kasprzak et al. 2003). Ni and its compounds have many industrial and commercial uses, and the progress of industrialization has led to increased emission of this environmental pollutant into our ecosystems. Ni is used in modern industry with other metals to form alloys to produce coins, jewelry, household and cooking utensils, batteries, orthopedic implants, and orthodontic appliances such as braces and dental appliances (Cempel and Nikel 2006). Environmental exposure to Ni by the general population occurs primarily via oral intake, as a contaminant in drinking water or as either a constituent or contaminant of foods, including chocolate, nuts, and grains (Cempel and Nikel 2006).

Occupational exposure to Ni occurs predominantly in the mining, refining, alloy production, electroplating, and welding industries (Doll et al. 1970; Grimsrud et al. 2000, 2002). Epidemiological studies of Ni compounds from occupationally exposed populations have reported that exposure to Ni compounds is associated with lung and nasal cancers and increased risks for acute respiratory syndromes, most clearly demonstrated in Ni refinery workers (Doll et al. 1970; Grimsrud et al. 2002; Rojas et al. 1999). In 1990, the International Agency for Research on Cancer (1990) classified Ni compounds as carcinogenic to both humans and experimental animals.

The eukaryotic genome is packaged into chromatin, the fundamental subunit of which is the nucleosome. Each nucleosome contains 146 bp DNA wrapped around an octamer of core histones. Two copies of each of histone, H2A, H2B, H3, and H4, form the histone core octamer.

Posttranslational modifications (i.e., acetylation, methylation, phosphorylation, ubiquitination) on the tails of the histone proteins play an important role in regulating chromatin biology (Turner 2007; Zhang and Reinberg 2001). These specific histone modifications and their combinations are translated, through protein interactions, into distinct effects on nuclear processes, such as activating or inhibiting transcription (Jenuwein and Allis 2001). Acetylation of lysines on histone tails is associated with transcriptional activation (Allfrey et al. 1964). Methylation of histones H3K4 and H3K36 is associated with actively transcribed genes, whereas methylation of H3K9, H3K27, and H4K20 is associated with transcriptional repression. Furthermore, lysine residues on histone tails can be mono-, di-, or trimethylated, and the degree of methylation on H3K4, H3K9, H3K27, and H4K20 has considerable influence on transcriptional activation or repression (Fuchs et al. 2006; Santos-Rosa et al. 2002; Tamaru et al. 2003; Zhang et al. 2007). For example, H3K4 trimethylation and H3K9 acetylation are generally associated with transcriptional activation, whereas H3K9 dimethylation is associated with transcriptional repression (Fuchs et al. 2006).

Ni compounds were found to have weak mutagenic activity in our laboratory (Biggart and Costa 1986; Klein et al. 1991). In other laboratories, nickel subsulfide and green nickel oxide did not induce mutation to ouabain resistance in C3H/10T12 mouse embryo cells, nor did nickel subsulfide induce mutation to ouabain resistance or to 6-thioguanine resistance in cultured human diploid fibroblasts, although crystalline nickel monosulfide and green nickel oxide did induce amplification of the ect-2 protooncogene in C3H/10T1/2 mouse embryo cells (Biedermann and Landolph 1987; Clemens et al. 2005; Miura et al. 1989). Therefore, Ni carcinogenesis likely proceeds through a combination of genetic (mutation and gene amplification) and epigenetic mechanisms (Biggart and Costa 1986; Clemens et al. 2005; Kang et al. 2003; Ke et al. 2006, 2008).

Previous studies have shown that *in vitro* exposure to Ni can perturb DNA methylation patterns as well as global and gene-specific levels of histone modifications. Ni-induced changes in histone modifications include loss of acetylation on histones H2A, H2B, H3, and H4and increases in histone H3K9me2 and H3K4me3, and ubiquitination of histones H2A and H2B (Chen et al. 2006; Kang et al. 2003; Ke et al. 2008; Zhou et al. 2009). However, the studies examining the changes in global and gene-specific DNA methylation patterns and histone modifications induced by exposure to Ni compounds have only been conducted in tissue culture model systems.

The present study was conducted in a Chinese population to determine whether occupational exposure to the Ni dusts from refining sulfidic Ni ores is associated with alterations in global levels of H3K4 trimethylation (leading to H3K4me3), H3K9 acetylation (leading to H3K9ac), and H3K9 dimethylation (leading to H3K9me2) histone modifications in peripheral blood mononuclear cells (PBMCs) of subjects. Additionally, the inter- and intraindividual variance of global levels of H3K4me3, H3K9ac, and H3K9me2 histone modifications in PBMCs of subjects was evaluated to determine if measurements of global levels of histone modifications are stable over time.

## Materials and Methods

*Study site and subject recruitment.* This study was conducted among workers of a Ni refinery in Jinchang, China, and local residents in Gansu, China. The human subject protocol for this study was approved by the institutional review boards of both the New York University School of Medicine and the Lanzhou University School of Public Health. Written informed consent was obtained from all participating subjects.

We recruited 120 healthy male subjects between 24 and 56 years of age for this study; subjects with diagnosed chronic diseases, including cancer, were excluded. For phase 1 of the study, 30 subjects who had high occupational exposure to Ni for at least 1 year at a Ni refinery in Jinchang, China, having worked in the flash smelting workshop where sulfidic Ni ores are processed, were recruited through questionnaire interview. Sixty referent subjects, who were maintenance or office workers, with no reported occupational exposure to Ni, were enrolled from residents in Gansu, China. The referents were frequency matched (2:1) by age and smoking habits with the 30 recruited occupationally exposed workers. For phase 2 of the study, 15 additional subjects with occupational exposure to Ni and 15 additional referent subjects were recruited, as in phase 1. The phase 1 study was conducted to determine whether exposure to Ni is associated with alterations in global levels of histone modification markers in the PBMCs of subjects. The phase 2 study was conducted to evaluate the inter- and intraindividual variability of global levels of histone modifications. Ni-exposed subjects are exposed to Ni ambient concentrations as high as 1 mg/m^3^. Referent subjects are exposed to Ni ambient concentrations of 204.8 ± 268.6 ng/m^3^.

*Sample collection and handling.* In the phase 1 study, a single blood and urine sample was collected. For phase 2, blood and urine samples were collected at three different time points, with a 1-week interval between collections. Blood samples (20 mL) were obtained by venipuncture by a local registered nurse. Blood was collected into two heparin-containing Vacutainer tubes, and all blood samples were temporarily kept at 4°C until transported to the local laboratory for isolation of PBMCs using a standardized Ficoll-Hypaque gradient procedure. Fifty microliters of urine was collected from each study subject. The isolated lymphocyte pellet and urine samples were stored frozen at –80°C and hand-carried frozen on dry ice to New York University.

*Histone extraction.* Histones were extracted from PBMCs as described previously (Chen et al. 2006) with a slight modification (Shechter et al. 2007). Briefly, cells were washed with ice-cold phosphate-buffered saline (PBS) and lysed in ice-cold radioimmunoprecipitation assay (RIPA) buffer (50 mM Tris-HCl, pH 7.4, 1% NP-40, 0.25% Na-deoxycholate, 150 mM NaCl, 1 mM EDTA, 1 mM phenylmethylsulfonyl fluoride, 1 µg/mL aprotinin, 1 µg/mL leupeptin, 1 µg/mL pepstatin, 1 mM Na_3_3VO_4_, 1 mM NaF) supplemented with a protease inhibitor mixture (Roche Applied Sciences, Indianapolis, IN, USA) for 10 min. The pellet was collected by centrifugation at 10,000 × *g* for 10 min. The pellet was washed once in 10 mM Tris-HCl and 37 mM EDTA (pH 7.4), and resuspended in 200 µL 0.4 N H_2_SO_4_. After overnight incubation on ice, the supernatant was collected by centrifugation at 14,000 × *g* for 15 min and mixed with 2 mL cold acetone and kept at −20°C overnight. The histones were collected by centrifugation at 14,000 × *g* for 15 min. After one wash with acetone, the histones were air dried and suspended in sterile deionized water. Total histone concentration in each sample was measured using the Bradford assay according to the manufacturer’s instructions (Bio-Rad Laboratories, Hercules, CA, USA).

**Figure 1:**
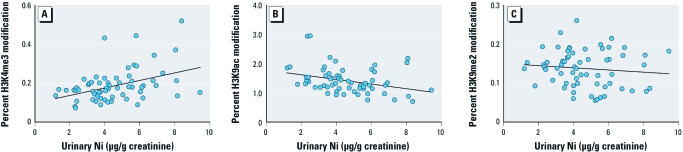
Association of urinary levels with global H3K4me3 (*y* = 0.02*x* + 0.1, *r* = 0.4, *p* = 0.0008) (*A*), H3K9ac (*y* = –0.08*x* + 1.8, *r* = 0.3, *p* = 0.0114) (*B*), and H3K9me2 (*y *= –0.003*x* + 0.2, *r* = 0.1, *p* = 0.4174) (*C*) from Ni-exposed and referent study subjects.

*Measurement of global histone modification.* Histone modification measurements were determined using a sandwich enzyme-linked immunosorbent (ELISA) assay. Polystyrene 96-well microplates (Fisher Scientific, Pittsburg, PA, USA) were coated with 100 µL of histone H3 antibody (Abcam, Cambridge, MA, USA) at a concentration of 1:20,000 diluted in PBS and incubated overnight at 4°C. The plates were washed with PBST (1× PBS, 0.05% Tween-20) and blocked for 2 hr at room temperature with 5% milk in PBST. The plates were washed with PBST and the desired amount of standard recombinant proteins (for measurement of the standard curve for H3K9me2 or H3K4me3; Active Motif, Carlsbad, CA, USA) or calf histone proteins (for measurement of the standard curve for H3K9ac; Sigma, Saint Louis, MO, USA) were added to each well, followed by the addition of PBMC histones diluted in water. Plates were incubated at room temperature for 1.5 hr with agitation on an orbital shaker. After incubation, the wells were washed with PBST, and 100 µL diluted primary antibody [histone H3 (Sigma), H3K9me2 (Abcam, Cambridge, MA, USA), H3K4me3 (Millipore, Billerica, MA, USA), or H3K9ac (Abcam)] was added to each well separately and incubated at room temperature for 1 hr with agitation. The wells were then washed with PBST, and 100 µL diluted secondary antibody (Santa Cruz Biotechnology, Santa Cruz, CA, USA) was added to each well and incubated at room temperature for 1 hr without agitation. Wells were washed with PBST and 100 µL of TMB (3,3´, 5,5˝-tetramethylbenzidine; Fisher Scientific) solution was added to each well and incubated at room temperature for 30 min in the dark. The reaction was stopped by adding 100 µL 2 M H_2_SO_4_ to each well. All analyses were performed in triplicate. The optical density was read at 450 nm using the SoftMax Pro software (version 5.2) and the SpectraMax 190 microplate reader (both from Molecular Devices, Sunnyvale, CA, USA). The relative percent histone modification was derived from standard curves specific to each histone modification. The respective within- and between-assay coefficients of variation for each modification were the following: H3K9me2, 3.9% and 6.6%; H3K9ac, 3.5% and 6.3%; and H3K4me3, 3.8% and 5.6%. Representative standard curves for H3, H3K4me3, H3K9ac, and H3K9me2 are provided in Supplemental Material [Figure 1 (http://dx.doi.org/10.1289/ehp.1104140)]. In phase 1, histone modifications for 23 Ni-exposed and 42 referent subjects were characterized. In phase 2, histone modifications for 15 Ni-exposed and 15 referent subjects were characterized. Measurements of global histone modifications were not measured for all subjects recruited into the study as proposed because of technical difficulties that occurred during the initial assay development.

*Measurement of urinary Ni, cotinine, and creatinine.* Urinary Ni, used to index the individual personal exposure to Ni, was analyzed for all study subjects by inductively coupled plasma mass spectroscopy (Elan DRCII; PerkinElmer, Norwalk, CT USA) (Oliveira et al. 2000). Urinary cotinine, a major metabolite of nicotine and a valid biomarker of environmental tobacco smoke, was measured in each subject to confirm smoking status and control its potential confounding effects. Urinary cotinine measurements were measured using a Cotinine Direct ELISA kit (Immunalysis, Pomona, CA, USA) (Hu et al. 2008). Urinary creatinine was measured in order to adjust the Ni and cotinine levels in urine samples. Urinary creatinine measurements were determined using the creatinine incorporating dynamic stabilization technology assay kits (Fisher Scientific) according to the standard procedure.

*Statistical analysis.* Differences in age, self-reported smoking data (number of smokers, number of years smoking, and cigarettes per day smoked for smokers), urinary cotinine, urinary Ni, and global levels of histone modifications among groups were compared by two-sample Student’s *t*-test. Differences in number of smokers and nonsmokers between exposure groups were compared by chi-square test. Linear regression analysis was used to evaluate the association of histone modification levels with urinary Ni levels. Multiple linear regression analysis was used to examine the association between urinary Ni and histone modification levels, while controlling for age and cigarette smoking as confounding factors. All *p*-values were two sided, with *p* < 0.05 considered statistically significant. Statistical analyses were performed in S-Plus statistical analysis software (TIBCO Software Inc., Palo Alto, CA, USA).

## Results

The phase 1 study of 30 subjects with high occupational exposure to Ni and 60 referents was designed to determine whether exposure to Ni induces alterations in global levels of histone modifications in PBMCs of subjects. As shown in Table 1, no significant difference was found between Ni-exposed and referent subjects with respect to age, self-reported data on smoking habits, or urinary cotinine levels. Although a large percentage of subjects reported that they were smokers in both groups, the Ni concentrations in blood plasma and urine are quite similar among smokers and nonsmokers (Torjussen et al. 2003). Urinary Ni was elevated in Ni-exposed subjects (5.68 ± 1.88 µg/g), compared with referents (3.96 ± 1.40 µg/g; *p* = 0.0006). H3K4me3 was elevated in the PBMCs of Ni-exposed subjects (0.25% ± 0.11%) compared with referents (0.15% ± 0.04%; *p* = 0.0004). H3K9me2 was decreased in the PBMCs of Ni-exposed subjects (0.11% ± 0.05%) compared with referents (0.15% ± 0.04%; *p* = 0.0027). H3K9ac did not differ significantly between groups (1.28% ± 0.55% for Ni-exposed subjects, 1.50% ± 0.36% for referents; *p* = 0.0981).

**Table 1:** Characteristics of the study subjects selected to study the alterations in histone modifications associated with occupational exposure to Ni (mean ± SD).

Parameter	Referent subjects*a*	Ni-exposed subjects*a*	*p*-Value
Age (years)		42.26 ± 6.19		43.74 ± 4.12		0.2626
Smoking (self-reported)						
Smokers [*n* (%)]		33 (79)		19 (83)		0.2601
Smoking years		17.03 ± 9.08		18.32 ± 6.79		0.5734
Cigarettes/day		14.73 ± 9.58		18.11 ± 8.52		0.2028
Urinary cotinine (µg/g creatinine)		8401.53 ± 9440.94		11517.86 ± 10049.98		0.2375
Urinary Ni (µg/g creatinine)		3.96 ± 1.40		5.68 ± 1.88		0.0006
Median		3.88		5.55		
Minimum		1.19		2.49		
Maximum		6.91		9.43		
10th percentile		2.30		3.54		
25th percentile		3.18		4.01		
75th percentile		4.73		7.07		
90th percentile		6.04		8.13		
Histone modifications (relative %)						
H3K4me3		0.15% ± 0.04		0.25% ± 0.11		0.0004
H3K9ac		1.50% ± 0.36		1.28% ± 0.55		0.0981
H3K9me2		0.15% ± 0.04		0.11% ± 0.05		0.0027
**a**Histone modifications for 42 referent subjects and 23 Ni-exposed subjects were characterized. Global histone modifications were not measured for all 60 referent subjects and 30 Ni-exposed subjects as proposed because of technical difficulties that occurred during the initial assay development.						

Linear regression analysis was used, combining data from Ni-exposed and referent subjects, to determine whether levels of urinary Ni were associated with the extent of H3K4me3, H3K9ac, and H3K9me2 histone modifications. H3K4me3 was positively associated (Figure 1A; *p* = 0.0008) and H3K9ac was negatively associated (Figure 1B; *p* = 0.0114) with urinary Ni, whereas H3K9me2 did not correlate with urinary Ni (Figure 1C; *p* = 0.4174).

Multiple regression analyses results indicated that age and cigarette smoke were not confounders for the association observed between urinary Ni and global histone modifications, with positive correlations between H3K4me3 and urinary Ni levels remaining after adjustment for age and self-reported smoking data (Table 2; *p* = 0.001) and for age and urinary cotinine levels (Table 2; *p* = 0.001). Also, a negative correlation was observed between urinary Ni and H3K9ac, after adjustment for age and self-reported smoking (Table 3; *p* = 0.01) and age and urinary cotinine levels (Table 3; *p* = 0.01). Correlations remained absent between H3K9me2 and urinary Ni levels when adjusted for age and self-reported smoking data (Table 4; *p* = 0.49) and for age and urinary cotinine levels (Table 4; *p* = 0.49).

**Table 2:** Summary of multiple regression analyses of global H3K4me3 on urinary Ni levels (µg/g) with adjustment of potential confounders.

Parameter	Estimate ± SE	*p*-Value
Without adjustment				
Intercept		0.99 ± 0.27		5 × 10^–4^
Urinary Ni		0.19 ± 0.05		8 × 10^–4^
Adjusted by age and smoking				
Intercept		1.12 ± 8.86		0.90
Urinary Ni		1.87 ± 0.55		1 × 10^–3^
Age		0.23 ± 0.18		0.22
Smoking (self-reported)		–0.79 ± 2.55		0.76
Adjusted by age and cotinine				
Intercept		–3.30 ± 7.84		0.68
Urinary Ni		1.86 ± 0.54		1 × 10^–3^
Age		0.28 ± 0.17		0.11
Urinary cotinine		1.64 × 10^–4^ ± 9.88 × 10^–5^		0.10


**Table 3:** Summary of multiple regression analyses of global H3K9ac on urinary Ni levels (µg/g) with adjustment of potential confounders.

Parameter	Estimate ± SE	*p*-Value
Without adjustment		
Intercept	1.77 ± 14.55	< 1 × 10^–4^
Urinary Ni	–7.73 ± 2.96	0.01
Adjusted by age and smoking		
Intercept	168.37 ± 48.22	9 × 10^–4^
Urinary Ni	–7.61 ± 2.99	0.01
Age	–0.07 ± 1.00	0.94
Smoking (self-reported)	14.77 ± 13.87	0.29
Adjusted by age and cotinine		
Intercept	175.78 ± 43.04	1 × 10^–4^
Urinary Ni	–7.68 ± 2.95	0.01
Age	–0.16 ± 0.95	0.87
Urinary cotinine	9.05 × 10^–4^ ± 5.42 × 10^–4^	0.10


**Table 4:** Summary of multiple regression analyses of global H3K9me2 on urinary Ni levels (µg/g) with adjustment of potential confounders.

Parameter	Estimate ± SE	*p*-Value
Without adjustment		
Intercept	15.11 ± 1.58	< 1 × 10^–4^
Urinary Ni	–0.26 ± 0.32	0.42
Adjusted by age and smoking		
Intercept	24.63 ± 5.13	< 1 × 10^–4^
Urinary Ni	–0.22 ± 0.32	0.49
Age	–0.20 ± 0.11	0.07
Smoking (self-reported)	–1.58 ± 1.48	0.29
Adjusted by age and cotinine		
Intercept	22.71 ± 4.67	0.0
Urinary Ni	–0.22 ± 0.32	0.50
Age	–0.17 ± 0.10	0.10
Urinary cotinine	–3.68 × 10^–5^ ± 5.88 × 10^–5^	0.53


For phase 2 of the study, we sought to evaluate the intraindividual variance (variance within subjects) compared with interindividual (variance between subjects) of global levels of H3K4me3, H3K9ac, and H3K9me2 in order to determine whether measurements of global levels of these histone modifications are relatively constant within subjects over time. The characteristics for participating subjects of phase 2 of the study are described in Table 5. As shown in Table 6, the variations of H3K4me3, H3K9ac, and H3K9me2 were substantially larger between subjects relative to the variations within subjects in both exposure groups, resulting in reliability coefficients (an estimate of the consistency of a set of measurements) of 0.60, 0.67, and 0.79 for H3K4me3, H3K9ac, and H3K9me2, respectively, for Ni-exposed subjects and 0.75, 0.74, and 0.97, respectively, for referent subjects. These results indicate that temporal variability within individuals is relatively small, compared with variability between subjects, suggesting that global H3K4me3, H3K9ac, and H3K9me2 histone modifications are relatively stable over time in human PBMCs from both Ni-exposed and referent subjects.

**Table 5:** Characteristics of the Ni-exposed and referent study subjects selected to study the intra- and interindividual variations of global H3K4me3, H3K9ac, and H3K9me2.

Parameter	Referent	Ni-exposed
Age		41.13 ± 8.36		41.40 ± 8.63
Smoking (self-reported)				
Smokers [*n* (%)]		8 (53)		8 (53)
Smoking years		20.88 ± 8.10		22.88 ± 6.60
Cigarettes/day		18.75 ± 13.92		21.88 ± 9.23
Urinary cotinine (µg/g creatinine)				
First collection		325.74 ± 104.78		258.53 ± 111.63
Second collection		341.92 ± 160.32		293.00 ± 148.93
Third collection		356.40 ± 161.35		314.39 ± 226.22
Urinary Ni (µg/g creatinine)				
First collection				
Mean ± SD		6.86 ± 1.88		6.67 ± 3.25
Median		7.01		5.96
Minimum		3.32		2.19
Maximum		10.42		12.03
10th percentile		4.99		3.41
25th percentile		5.49		4.27
75th percentile		8.09		9.12
90th percentile		8.77		10.29
Second collection				
Mean ± SD		6.51 ± 2.75		10.47 ± 4.40
Median		5.73		9.96
Minimum		3.45		6.11
Maximum		13.47		21.97
10th percentile		3.80		6.73
25th percentile		4.88		7.10
75th percentile		7.86		11.45
90th percentile		9.47		15.02
Third collection				
Mean ± SD		9.63 ± 2.91		8.84 ± 4.58
Median		9.48		8.02
Minimum		4.73		5.34
Maximum		15.00		23.65
10th percentile		6.69		5.54
25th percentile		7.51		6.44
75th percentile		11.11		9.40
90th percentile		13.45		11.25
Values are mean ± SD except where indicated.				

**Table 6:** Intra- and interindividual variability of global H3K4me3, H3K9ac, and H3K9me2 in human PBMCs of Ni-exposed and referent study subjects.

Parameter	Variance between subjects	Variance within subjects	Reliability coefficient (*R*)*a*
H3K4me3 (%)						
Referent subjects		3 × 10^–5^		1 × 10^–5^		0.75
Ni-exposed subjects		3 × 10^–5^		2 × 10^–5^		0.60
H3K9ac (%)						
Referent subjects		14.64		5.29		0.74
Ni-exposed subjects		36.69		14.93		0.67
H3K9me2 (%)						
Referent subjects		4.54 × 10^–3^		1.5 × 10^–4^		0.97
Ni-exposed subjects		5.86 × 10^–3^		1.53 × 10^–3^		0.79
Urinary Ni (µg/g creatinine)						
Referent subjects		7.21		5.68		0.56
Ni-exposed subjects		16.53		10.98		0.60
**a**Reliability coefficient (*R*) = *variance*_between_/(*variance*_between_ + *variance*_within_).						

## Discussion

In this first study evaluating histone modifications in relation to occupational Ni exposure in refinery workers and to human urinary Ni levels, we found highly significant elevated levels of H3K4me3 modification in Ni-exposed workers and in subjects with greater urinary Ni, consistent with previous studies reporting that exposure to Ni increases global levels of H3K4me3 *in vitro* (Zhou et al. 2009). H3K9ac modifications were marginally (and not significantly) decreased in Ni-exposed subjects; however, these modifications were significantly decreased in relation to greater urinary Ni. Consistent with the negative association found between H3K9ac and urinary Ni, previous *in vitro* studies found that exposure to Ni induces histone hypoacetylation of all four core histones (Kang et al. 2003). We found lower levels of H3K9me2 in subjects with occupational exposure to Ni; however, urinary Ni was not clearly associated with modifications in this histone. These results are not consistent with a previous report that *in vitro* exposure to Ni induces an increase in global H3K9me2 (Chen et al. 2006).

Plausible mechanisms by which exposure to Ni compounds induce alterations of global levels of histone modification *in vivo* include Ni-induced inhibition of histone demethylase and acetyltransferase enzymes. In support of this, previous studies from our group examining the mechanisms by which Ni compounds alter histone modifications *in vitro* have revealed that exposure to Ni decreases histone acetylation by inhibiting histone acetyltransferase activity, but has no effect on histone deacetylases (Kang et al. 2003). We have also shown that Ni increases H3K9me2 by inhibiting the demethylating activity, by directly binding and replacing the iron(II) from the enzyme JHDM2A/JMJD1A (KDM3A), with no observable effect on methyltransferases (Chen et al. 2006, 2010a, 2010b). We also recently reported that hypoxia increases H3K4me3 by inhibition of JARID1A demethylase, and because Ni is a hypoxia mimetic, it is possible that Ni also increases H3K4me3 levels by inhibiting the JARID1A demethylase or other members of the JARID1 family (Zhou et al. 2010).

It was not surprising that exposure to Ni increases histone modifications associated with gene repression decrease in H3K9ac, and also increase H3K4me3, associated with gene activation, because changes observed in previous *in vitro* studies (Chen et al. 2006, 2010a; Zhou et al. 2009) also suggest that induction of both gene activating and repressive histone modifications occur as a result of exposure to specific metal compounds. Although both H3K9me2 and H3K4me3 increased after exposures to Ni, arsenic, and chromate, distinct localization of chromate-induced H3K9me2 and H3K4me3 modifications in chromatin were detected with dual immunofluorescence: H3K9me2 was primarily enriched at the periphery of the nucleus, which coincides with the location of heterochromatin, whereas H3K4me3 was exclusively enriched in the center of the nucleus (euchromatin) (Zhou et al. 2009).

In a study of healthy workers at a steel plant in Brescia, Italy, Cantone et al. (2011) observed that global H3K4me2 was increased in association with Ni, arsenic, and iron, whereas H3K9ac was positively but not significantly associated with Ni and iron; results for H3K4me3 and H3K9me2 were not reported. The different results in our study may be explained by differences in the type of exposure of the subjects. Ni exposure in a steel plant is associated with exposures to other metals and in that case Ni exposure might track with other measured and unmeasured exposures, which may also affect levels of histone modification. Furthermore, both studies are relatively small, indicating the need for more detailed investigations in humans of metal and epigenetic modifications. Extension of studies will also need to consider whether changes in global levels of histone modifications occur in normal or malignant lung tissue exposed to Ni *in vivo*.

Our study had the advantage of investigating histone modifications in Ni-exposed subjects who worked in a Ni refinery, a setting known for excess risk of lung cancer and not notably confounded by other metal exposures. We also had the advantage of including a urinary biomarker for exposure, showing that Ni effects may occur under both occupational and environmental conditions. Because urinary Ni levels represent short-term exposure to Ni, global levels of H3K4me3 or H3K9ac histone modifications may potentially serve as biomarkers of exposure to Ni compounds. Further supporting our approach, we showed that single measurements of global H3K4me3, H3K9ac, and H3K9me2 histone modifications are representative of modification status over time.

Our study points to epigenetic control of carcinogenesis in Ni-exposed humans and points to alterations in global levels of histone modification in subjects with Ni occupational exposure comparable to limits for Ni exposures in the United States (Occupational Safety and Health Administration permissible exposure limit, 0.1 mg/m^3^ for water-soluble Ni and 1.0 mg/m^3^ for Ni subsulfide, oxide, and metallic), Canada (time-weighted average exposure values of 1.0 mg/m^3^ for Ni metal, oxides, and sulfides and 0.1 mg/m^3^ for water-soluble Ni compounds), Germany (0.5 mg/m^3^ for metallic and sulfide Ni, sulfidic ores, Ni oxide, and Ni carbonate), and the United Kingdom (maximum exposure limits of 0.5 mg/m^3^ for Ni metal and water-insoluble Ni compounds and 0.1 mg/m^3^ for water-soluble Ni). The ambient levels of environmental exposure to Ni by referent subjects are also comparable to ambient levels in the United States (120–170 ng/m^3^ in industrialized regions and large cities).

An increasing number of human diseases have been associated with aberrant epigenetic alterations, including cancer and cardiovascular, neurological, and autoimmune disorders (Wang et al. 2009). Because changes in epigenetic marks are associated with a wide range of diseases, epigenetic therapies are attractive options for risk profiling and treatment of specific disorders. Therefore, deciphering epigenetic markers in humans is crucial because the scope of epigenetic therapies is likely to expand in the coming years (Peedicayil 2006).

## Conclusions

Further studies should be directed toward determining whether the alterations in histone modifications induced by exposure to Ni identified in the present study are associated with risk of cancer, as well as identifying changes in gene expression and biochemical pathways affected as a result of exposure to Ni compounds. The results of this study suggest the possible use of global levels of histone modifications as biomarkers of exposure to Ni compounds. Well-characterized histone modifications may aid in the future in the diagnosis and treatment of a variety of cancers or other disease states.

## Supplemental Material

(172 KB) PDF

## References

[r1] Allfrey VG, Faulkner R, Mirsky AE (1964). Acetylation and methylation of histones and their possible role in the regulation of RNA synthesis.. Proc Natl Acad Sci USA.

[r2] Biedermann KA, Landolph JR (1987). Induction of anchorage independence in human diploid foreskin fibroblasts by carcinogenic metal salts.. Cancer Res.

[r3] Biggart NW, Costa M (1986). Assessment of the uptake and mutagenicity of nickel chloride in Salmonella tester strains.. Mut Res.

[r4] Cantone L, Nordio F, Hou L, Apostoli P, Bonzini M, Tarantini L (2011). Inhalable metal-rich air particles and histone H3K4 dimethylation and H3K9 acetylation in a cross-sectional study of steel workers.. Environ Health Perspect.

[r5] Cempel M, Nikel G. (2006). Nickel: a review of its sources and environmental toxicology.. Pol J Environ Stud.

[r6] Chen H, Giri NC, Zhang R, Yamane K, Zhang Y, Maroney M (2010a). Nickel ions inhibit histone demethylase JMJD1A and DNA repair enzyme ABH2 by replacing the ferrous iron in the catalytic centers.. J Biol Chem.

[r7] Chen H, Ke Q, Kluz T, Yan Y, Costa M. (2006). Nickel ions increase histone H3 lysine 9 dimethylation and induce transgene silencing.. Mol Cell Biol.

[r8] Chen H, Kluz T, Zhang R, Costa M. (2010b). Hypoxia and nickel inhibit histone demethylase JMJD1A and repress Spry2 expression in human bronchial epithelial BEAS-2B cells.. Carcinogenesis.

[r9] Clemens F, Verma R, Ramnath J, Landolph JR (2005). Amplification of the Ect2 proto-oncogene and over-expression of Ect2 mRNA and protein in nickel compound and methylcholanthrene-transformed 10T1/2 mouse fibroblast cell lines.. Toxicol Appl Pharmacol.

[r10] Doll R, Morgan LG, Speizer FE (1970). Cancers of the lung and nasal sinuses in nickel workers.. Br J Cancer.

[r11] Fuchs J, Demidov D, Houben A, Schubert I. (2006). Chromosomal histone modification patterns—from conservation to diversity.. Trends Plant Sci.

[r12] Grimsrud TK, Berge SR, Haldorsen T, Andersen A (2002). Exposure to different forms of nickel and risk of lung cancer.. Am J Epidemiol.

[r13] Grimsrud TK, Berge SR, Resmann F, Norseth T, Andersen A (2000). Assessment of historical exposures in a nickel refinery in Norway.. Scand J Work Environ Health.

[r14] Hu Y, Li G, Xue X, Zhou Z, Li X, Fu J (2008). PAH-DNA adducts in a Chinese population: relationship to PAH exposure, smoking and polymorphisms of metabolic and DNA repair genes.. Biomarkers.

[r15] International Agency for Research on Cancer (1990). Chromium, Nickel and Welding.. IARC Monogr Eval Carcinog Risks Hum.

[r16] Jenuwein T, Allis CD (2001). Translating the histone code.. Science.

[r17] Kang J, Zhang Y, Chen J, Chen H, Lin C, Wang Q (2003). Nickel-induced histone hypoacetylation: the role of reactive oxygen species.. Toxicol Sci.

[r18] Kasprzak KS, Sunderman FW, Salnikow K (2003). Nickel carcinogenesis.. Mutat Res.

[r19] Ke Q, Davidson T, Chen H, Kluz T, Costa M. (2006). Alterations of histone modifications and transgene silencing by nickel chloride.. Carcinogenesis.

[r20] Ke Q, Ellen TP, Costa M (2008). Nickel compounds induce histone ubiquitination by inhibiting histone deubiquitinating enzyme activity.. Toxicol Appl Pharmacol.

[r21] Klein CB, Conway K, Wang XW, Bhamra RK, Lin XH, Cohen MD (1991). Senescence of nickel-transformed cells by an X chromosome: possible epigenetic control.. Science.

[r22] Miura T, Patierno SR, Sakuramoto T, Landolph JR (1989). Morphological and neoplastic transformation of C3H/10T1/2 Cl 8 mouse embryo cells by insoluble carcinogenic nickel compounds.. Environ Mol Mutagen.

[r23] Oliveira JP, de Siqueira ME, da Silva CS (2000). Urinary nickel as bioindicator of workers’ Ni exposure in a galvanizing plant in Brazil.. Int Arch Occup Environ Health.

[r24] Peedicayil J. (2006). Epigenetic therapy—a new development in pharmacology.. Indian J Med Res.

[r25] Rojas E, Herrera LA, Poirier LA, Ostrosky-Wegman P (1999). Are metals dietary carcinogens?. Mutat Res.

[r26] Santos-Rosa H, Schneider R, Bannister AJ, Sherriff J, Bernstein BE, Emre NC (2002). Active genes are tri-methylated at K4 of histone H3.. Nature.

[r27] Shechter D, Dormann HL, Allis CD, Hake SB (2007). Extraction, purification and analysis of histones.. Nat Protoc.

[r28] Tamaru H, Zhang X, McMillen D, Singh PB, Nakayama J, Grewal SI (2003). Trimethylated lysine 9 of histone H3 is a mark for DNA methylation in Neurospora crassa.. Nat Genet.

[r29] Torjussen W, Zachariasen H, Andersen I. (2003). Cigarette smoking and nickel exposure.. J Environ Monit.

[r30] Turner BM (2007). Defining an epigenetic code.. Nat Cell Biol.

[r31] Wang Z, Schones DE, Zhao K (2009). Characterization of human epigenomes.. Curr Opin Genet Dev.

[r32] Zhang X Clarenz O Cokus S Bernatavichute YV Pellegrini M Goodrich J 2007 Whole-genome analysis of histone H3 lysine 27 trimethylation in *Arabidopsis*. PLoS Biol 5 5 e129; doi:10.1371/journal.pbio.0050129 [Online 17 April 2007] 17439305 PMC1852588

[r33] Zhang Y, Reinberg D. (2001). Transcription regulation by histone methylation: interplay between different covalent modifications of the core histone tails.. Genes Dev.

[r34] Zhou X, Li Q, Arita A, Sun H, Costa M. (2009). Effects of nickel, chromate, and arsenite on histone 3 lysine methylation.. Toxicol Appl Pharmacol.

[r35] Zhou X, Sun H, Chen H, Zavadil J, Kluz T, Arita A (2010). Hypoxia induces trimethylated H3 lysine 4 by inhibition of JARID1A demethylase.. Cancer Res.

