# Supplementary material for: Global Levels of Histone Modifications in Peripheral Blood Mononuclear Cells of Subjects with Exposure to Nickel
Source: Environ Health Perspect. 2011 Oct 24;120(2):198–203. doi: 10.1289/ehp.1104140 (PMC3279455; doi:10.1289/ehp.1104140)
Supplement: (172 KB) PDF [file ehp.1104140.s001.pdf]

Supplemental Material for Environmental Health Perspectives

# Global Levels of Histone Modifications in Peripheral Blood Mononuclear Cells of Subjects with Exposure to Nickel

Adriana Arita[1], Jingping Niu[2], Qingshan Qu[1], Najuan Zhao[2], Ye Ruan[2], Arthur Nadas[1], Yana Chervona[1], Fen Wu[1], Hong Sun[1], Richard B. Hayes[1,3], Max Costa[1,3*]

[1]New York University School of Medicine, Department of Environmental Medicine, New York, U.S.A

[2]Lanzhou University School of Public Health, Lanzhou, China

[3]New York University Cancer Institute, New York, U.S.A.

[*]Address correspondence to Max Costa, New York University, Department of Environmental Medicine, 57 Old Forge Road, Tuxedo, NY 10987, Tel. (845) 731-3515, Fax: (845) 731-2118, Max.Costa@nyumc.org

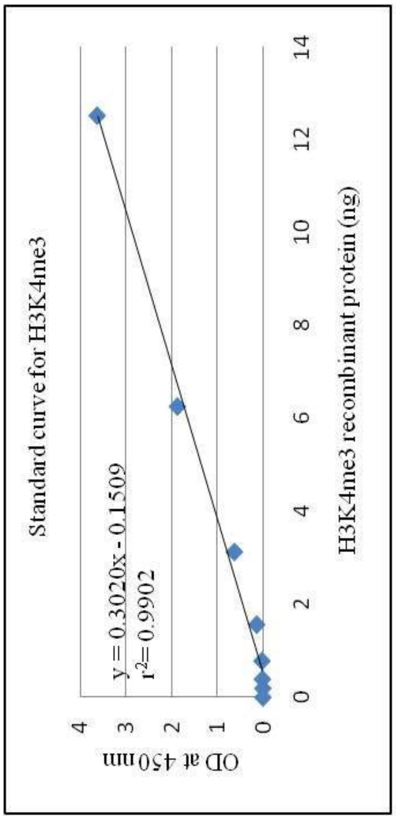
Standard curve for H3K4me3
y = 0.3020x - 0.1509
r2= 0.9902
OD at 450nm
H3K4me3 recombinant protein (ng)
0
1
2
3
4
0
2
4
6
8
10
12
14

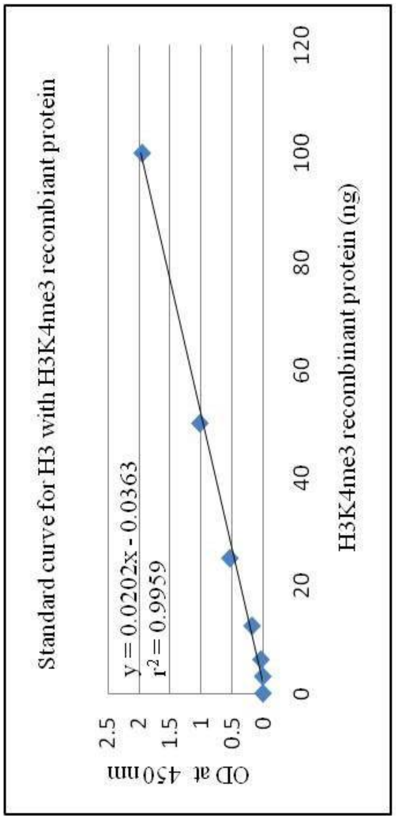
Standard curve for H3 with H3K4me3 recombiant protein
y = 0.0202x - 0.0363
r2 = 0.9959
OD at 450nm
H3K4me3 recombinant protein (ng)
0
0.5
1
1.5
2
2.5
0
20
40
60
80
100
120

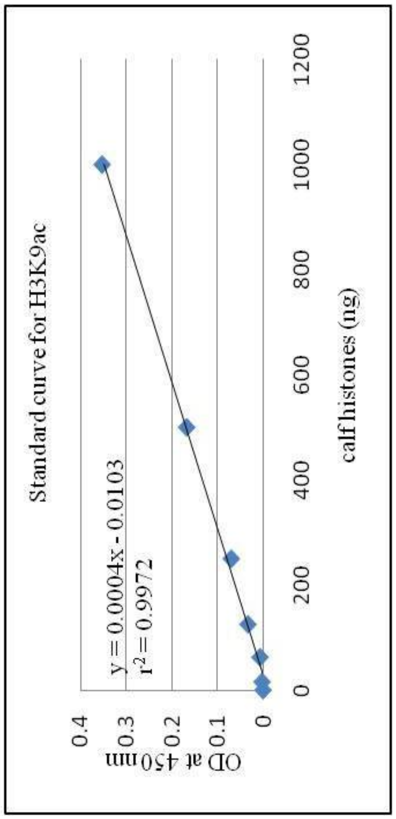
Standard curve for H3K9ac
y = 0.0004x - 0.0103
r2 = 0.9972
OD at 450nm
calf histones (ng)
0
0.1
0.2
0.3
0.4
0
200
400
600
800
1000
1200

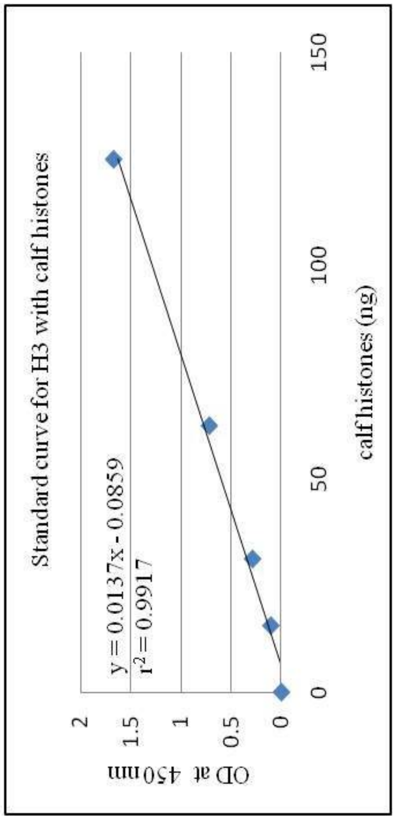
Standard curve for H3 with calf histones
y = 0.0137x - 0.0859
r2 = 0.9917
OD at 450nm
calf histones (ng)
0
0.5
1
1.5
2
0
50
100
150

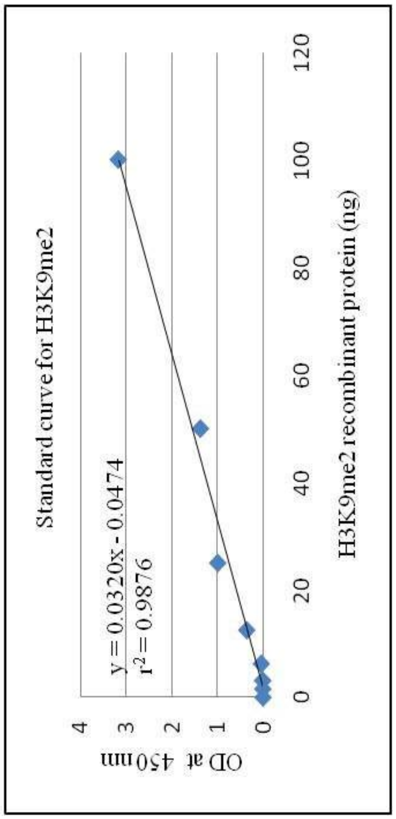
Standard curve for H3K9me2
y = 0.0320x - 0.0474
r2 = 0.9876
OD at 450nm
H3K9me2 recombinant protein (ng)
0
1
2
3
4
0
20
40
60
80
100
120

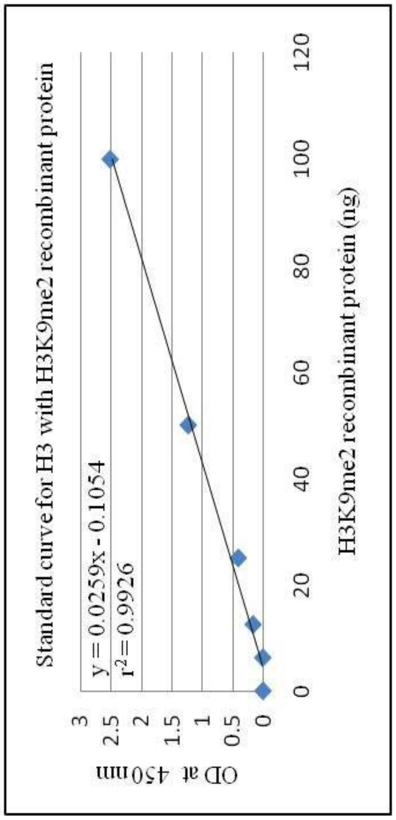
Standard curve for H3 with H3K9me2 recombinant protein
y = 0.0259x - 0.1054
r2 = 0.9926
OD at 450nm
H3K9me2 recombinant protein (ng)
0
0.5
1
1.5
2
2.5
3
0
20
40
60
80
100
120

**Supplemental Material, Figure 1.** Standard curves for measurement of global levels of H3K4me3, H3K9ac, and H3K9me2 histone modifications with enzyme-linked immunoabsorbent (ELISA) assay. Standard curves for H3K4me3 and H3K9me2 were calculated with H3K4me3 or H3K9me2 recombinant proteins (Active Motif, Carlsbad, CA). Recombinant Xenopus Laevis proteins are produced in *E.coli* and purified using Fast protein liquid chromatography (FPLC). The purified proteins are substantially alkylated to specifically trimethylate H3K4me3 or dimethylate H3K9 and repurified prior to lyophilization. The degree of methylation is >99% for H3K4me3 and H3K9me2 recombinant protein. Samples for standard curves for H3K9ac were prepared with calf histone proteins (Sigma, Saint Louis, MO). Standard curves for H3K4me3 were prepared with serial dilutions beginning with 12.5 ng of the recombinant protein. Standard curves for H3K9me3 and H3K9ac were prepared with serial dilutions beginning with 100 ng of recombinant protein or 1000 ng calf histone proteins, respectively. To calculate histone loading, standard curves for H3 were also prepared with the H3K4me3 or H3K9me2 recombinant proteins or calf histones. Standard curves for H3 for H3K4me3 or H3K9me2 were prepared with serial dilutions beginning with 100 ng H3K4me3 or H3K9me2 recombinant protein. Standard curves for H3 for H3K9ac were prepared with serial dilutions beginning with 125 ng calf histones. All standard curves were performed with concentrations up to where the conditions were linear.
